# Transforming research education for translational science and implementation of evidence-based practices

**DOI:** 10.1186/1748-5908-10-S1-A36

**Published:** 2015-08-20

**Authors:** Donna L Burton, Tom O Massey, Bruce Lubotsky Levin, Julie Baldwin, Heather Williamson

**Affiliations:** 1 University of South Florida, Tampa, FL, 33612, USA

## Statement of the problem

Children's mental health researchers have made great gains in demonstrating the effectiveness of interventions that result in desirable outcomes for children with behavioral health issues. However, despite proven efficacy, empirically supported treatments are not widely adopted in community practice settings. There is a growing body of evidence in support of guiding principles for participatory research and, at its core is the development of academic-community partnerships for more effective health disparities research.

## Research objectives

This presentation will discuss the Institute for Translational Research in Adolescent Behavioral Health (ITR), a NIDA funded comprehensive research education and training program for graduate students and community-based service providers. The inclusion of a service learning component and community partners that span a range of services relevant to child and adolescent behavioral health ensures that the education program maintains a fully integrated research-to-practice platform. As such, ITR provides a unique multidisciplinary approach and a timely example of the emphasis on academic-community partnerships for translational research.

## Methods

Surveys of trainees, along with interviews of trainees, academic mentors, and community service partners were used to assess the effectiveness of the Institute fostering the development of behavioral health researchers and practitioners, promoting interdisciplinary research, and providing opportunities for trainees to apply the skills of research in the areas of translational research, and implementation of evidence-based practices. Preliminary outcomes from the first cohort of trainees will be discussed, and include insights, expectations, and experiences from trainees, factors associated with enrollment and feedback from community service partners on the progress of trainees.

## Relevance to dissemination and implementation research

The Institute is well positioned to become a model for transforming research education such that the fundamental skills of research are more relevant to the emerging field of dissemination and implementation science.

